# The Lung Screen Uptake Trial (LSUT): protocol for a randomised controlled demonstration lung cancer screening pilot testing a targeted invitation strategy for high risk and ‘hard-to-reach’ patients

**DOI:** 10.1186/s12885-016-2316-z

**Published:** 2016-04-20

**Authors:** Samantha L. Quaife, Mamta Ruparel, Rebecca J. Beeken, Andy McEwen, John Isitt, Gary Nolan, Karen Sennett, David R. Baldwin, Stephen W. Duffy, Samuel M. Janes, Jane Wardle

**Affiliations:** Health Behaviour Research Centre, Department of Epidemiology and Public Health, University College London, Gower Street, London, WC1E 6BT UK; Lungs for Living Research Centre, UCL Respiratory, Division of Medicine, Rayne Building, University College London, 5 University Street, London, WC1E 6JF UK; Resonant Media, 55 Old Compton Street, London, W1D 6HW UK; Killick Street Health Centre, 75 Killick Street, London, N1 9RH UK; Respiratory Medicine Unit, David Evans Research Centre, Nottingham University Hospitals, City Campus, Nottingham, NG5 1 PB UK; Wolfson Institute of Preventive Medicine, Barts and the London School of Medicine and Dentistry, Queen Mary University of London, Charterhouse Square, London, EC1M 6BQ UK

**Keywords:** Lung cancer, Cancer screening, Smoking, Health inequalities

## Abstract

**Background:**

Participation in low-dose CT (LDCT) lung cancer screening offered in the trial context has been poor, especially among smokers from socioeconomically deprived backgrounds; a group for whom the risk-benefit ratio is improved due to their high risk of lung cancer. Attracting high risk participants is essential to the success and equity of any future screening programme. This study will investigate whether the observed low and biased uptake of screening can be improved using a targeted invitation strategy.

**Methods/design:**

A randomised controlled trial design will be used to test whether targeted invitation materials are effective at improving engagement with an offer of lung cancer screening for high risk candidates. Two thousand patients aged 60–75 and recorded as a smoker within the last five years by their GP, will be identified from primary care records and individually randomised to receive either intervention invitation materials (which take a targeted, stepped and low burden approach to information provision prior to the appointment) or control invitation materials. The primary outcome is uptake of a nurse-led ‘lung health check’ hospital appointment, during which patients will be offered a spirometry test, an exhaled carbon monoxide (CO) reading, and an LDCT if eligible. Initial data on demographics (i.e. age, sex, ethnicity, deprivation score) and smoking status will be collected in primary care and analysed to explore differences between attenders and non-attenders with respect to invitation group. Those who attend the lung health check will have further data on smoking collected during their appointment (including pack-year history, nicotine dependence and confidence to quit). Secondary outcomes will include willingness to be screened, uptake of LDCT and measures of informed decision-making to ensure the latter is not compromised by either invitation strategy.

**Discussion:**

If effective at improving informed uptake of screening and reducing bias in participation, this invitation strategy could be adopted by local screening pilots or a national programme.

**Trial registration:**

This study was registered with the ISRCTN (International Standard Registered Clinical/soCial sTudy Number : ISRCTN21774741) on the 23^rd^ September 2015 and the NIH ClinicalTrials.gov database (NCT0255810) on the 22^nd^ September 2015.

**Electronic supplementary material:**

The online version of this article (doi:10.1186/s12885-016-2316-z) contains supplementary material, which is available to authorized users.

## Background

Worldwide, lung cancer kills more people than any other cancer, explaining over one fifth of all cancer-related mortality in the UK [1, 2]. Five-year survival is poor at just 11.1 % for men and 15.0 % for women [3], but prognosis improves significantly with earlier stage at diagnosis. For example, five-year survival estimates increase to 58–73 % when non-small cell lung cancer (NSCLC) is diagnosed at the earliest stage (stage 1A) [4]. However, close to 70 % of patients are diagnosed with advanced stage disease [5] with around 40 % presenting via emergency admission [6] and almost a third dying within 90 days of their diagnosis [7]. This is partly because detecting lung cancer early is challenging; early symptoms are typically non-specific and they may not even be manifest until the disease has progressed.

Data from the National Lung Screening Trial (NLST) suggest that screening individuals at high risk of lung cancer using low-dose computed tomography (LDCT) scans is a potential early detection strategy. A 20 % relative risk reduction in lung cancer mortality and a 6.7 % reduction for all-cause mortality was observed for patients aged 55–74 with a significant (≥30 pack-years) and recent (within 15 years) smoking history, who underwent three annual LDCT screens compared with chest X-rays [8]. Subsequently, the US Preventive Services Task Force (USPSTF) issued a grade B recommendation for screening high risk adults; a preventive service benefit now covered by Medicare and Medicaid Services [9]. The case for implementation is building within the UK where the National Screening Committee is due to make a decision by 2016, following results expected from the European trials.

For any screening programme to be effective, it must achieve a positive benefit-harm ratio, which in turn depends upon attracting the high risk population. Increasing the risk profile of participants has potential to reduce avoidable invasive follow-up tests and the number needed to screen [10]. Indeed, NLST participants categorised within the three highest quintiles of risk benefitted from 88 % of screen-prevented deaths [11]. However, enrolment to screening offered within the trial context has been extremely low, ranging from 0.2–4.6 % of the total age-eligible population invited [12–15], and biased toward former smokers, rather than current smokers, and towards higher socioeconomic status (SES) individuals [16, 17]. In the UK Lung Screening Trial (UKLS), the proportion of individuals with a high lung cancer risk score (using the Liverpool Lung Project model) [18] increased with socioeconomic deprivation, yet paradoxically response rates and subsequent clinic attendance decreased [15]. This suggests that despite their high risk, lower SES smokers are less likely to engage with an offer of screening or see it through; a pervasive problem observed across other screening programmes [19–21] and healthcare services [22, 23].

It is essential that screening communication effectively engages this group if lung cancer screening is to be an equitable early detection strategy and attain adequate uptake. To date, methods of recruitment into trials have been heterogeneous, including mass-mailing, media advertisements, community outreach and GP enrolment (e.g. [12, 14, 24]). Some initially invited all individuals in the at-risk age group who were requested to complete risk assessment measures and engage in further correspondence to determine eligibility. Therefore, while we know uptake is poorer among low SES smokers, it is difficult to ascertain the denominator of eligible individuals invited to screening needed to reliably calculate levels of uptake among high risk candidates. Furthermore, these individuals have been invited to participate in a research trial evaluating the clinical effectiveness of LDCT screening; an invitation that is likely to be interpreted very differently from that for a lung cancer screening service. To our knowledge, no study has taken a targeted approach to the design of invitation and information materials for (and in consultation with) high risk and ‘hard-to-reach’ groups, nor attempted to test such a strategy in the real-world context of a demonstration pilot lung cancer screening service.

### Aims

The primary aim of this study is evaluate the impact of a targeted invitation strategy, compared with a control, on uptake of ‘lung health check’ appointments overall and in association with demographic and smoking characteristics.

The secondary aims of this study are to:

1. compare the demographic and smoking-related characteristics of attenders versus non-attenders for each invitation group, and with the overall invited group,
2. explore informed decision-making outcomes by invitation group to check that the information provided to each is equally effective in facilitating a patient’s ability to make an informed decision at the appointment,
3. ascertain figures to help gauge uptake of a national screening programme and inform the feasibility of recruiting to a LDCT programme via primary care.

## Methods/design

### Study design

**Fig. 1:**
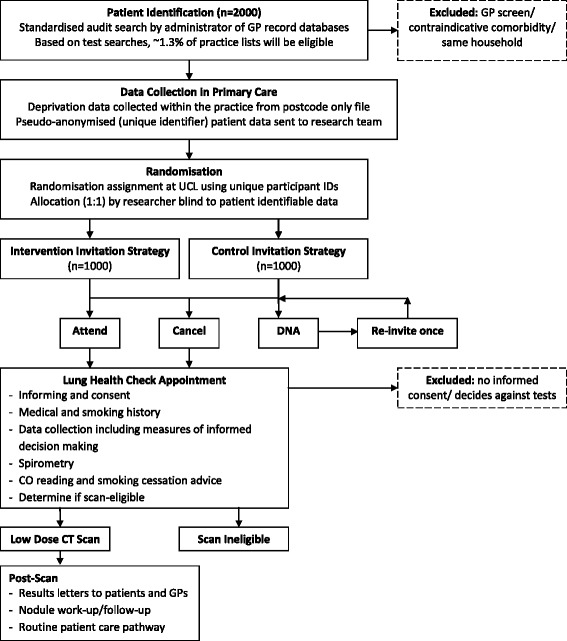
Trial flow diagram

This study will use a two-arm, between-subjects, individually-randomised controlled trial design to compare uptake of lung cancer screening appointments between two groups allocated to receive either intervention or control invitation materials (see Fig. 1 for an overview of participant flow through the trial).

### Randomisation and allocation procedure

The individual unit of randomisation will be the patient. A web-based randomisation programme has been constructed by an independent health research unit. This will randomise patients at a ratio of 1:1 using permuted blocks for each GP practice to ensure group allocation is evenly balanced by practice. Patient identifiable details will be concealed from the researcher carrying out the randomisation assignment using a pseudo-anonymised spreadsheet of eligible patient details exported securely to the researcher from the GP practice. Patients will be blind to their allocation and the research nature of the study, which would undermine the primary outcome.

### Setting and participants

Patients will be identified from primary care practices falling within three Clinical Commissioning Groups (CCGs): Islington, Camden, and City and Hackney. These sites were chosen because they have demographically diverse patient populations. All patients will be invited by their GP (by letter, including a clinic telephone helpline) to a pre-scheduled ‘lung health check’ appointment with two weeks’ notice. This will include an eligibility screen (i.e. smoking and medical history), spirometry test, CO reading, smoking cessation advice (for current smokers), and for those eligible, a LDCT scan. The appointments will be run by research nurses in outpatient clinics at a central London tertiary referral hospital and an inner London district general hospital (University College Hospital and the Homerton University Hospital). Informed consent will be taken by a research nurse at the beginning of each lung health check appointment who will explain that the purpose of the pilot is to measure uptake and will describe all other data being collected. Data on secondary outcomes will not be collected for patients who do not give consent.

### Eligibility criteria

#### Inclusion criteria

Patients will be eligible for invitation if they are aged 60–75 years and have been recorded by their GP practice as a current smoker at any point since April 2010. This threshold was chosen for two reasons: i) to identify a group likely to have accrued the 30 pack-year history conferring likely screening eligibility, and ii) to identify predominantly current smokers as this is the group most difficult to attract to screening.

#### Exclusion criteria

Patients will be excluded if they fulfil any of the following criteria: have an active lung cancer diagnosis or metastases, are on the palliative care register, have had a recent CT thorax (≤12 months), lack capacity to consent, or GP deems them unsuitable due to a comorbidity contraindicative of screening for lung cancer or subsequent treatment.

### Patient identification

The patient identification process will be supported during an initial site visit by a member of the research team. A standardised audit search will be imported and run by practice administrators to extract details of eligible patients from GP record databases with ease and consistency. The subsequent list of potentially eligible patients will then be screened by GPs for patients they deem unsuitable. To avoid contamination, only one eligible patient per household will be enrolled.

### Invitation procedure and adherence

The printing and mailing of materials will be carried out via a secure third party company on behalf of each GP practice. A researcher will support practice administrators in uploading patient details, specifying the contents of mail packs and assigning mailing dates using the company’s electronic system. Allocation of appointments will have been carried out by the research team at the randomisation stage and input into the spreadsheet of patient details so that these automatically populate the invitation letters. This in-practice assistance will also allow monitoring of adherence to the mailing protocol. The mailing company’s activity will also be monitored via checking of reported mailings to ensure they are being sent as instructed.

### Control invitation materials

Table 1 provides a detailed breakdown of the content, delivery and staging of information by invitation group. Invitations in both arms will be from the patient’s own GP. In the absence of ‘usual care’ invitation materials, control invitations will mimic so far as possible the best available materials and methods of established cancer screening programmes. These comprise the following:

**Table 1:** Information content and delivery by stage and invitation group

	Delivery	Content	
	(mode, messenger, recipients, time)	Control arm	Intervention arm
Pre-invitation	Mailed 3–4 weeks prior to the appt From GP (signature and letterhead) All patients	Pre-invitation letter notifying the patient of the lung health check service Information booklet mimicking so far as possible ‘the facts’ booklets for cancer screening programmes	Identical pre-invitation letter to control Targeted information leaflet introducing the tests using a low burden approach including: - content designed to reduce fear, fatalism, stigma - explanation and diagram to show how early treatment can work - quotes from interview participants to address stigma and highlight benefit - emphasis on non-judgemental service
Invitation	Mailed 2–3 weeks prior to the appt From GP (signature and letterhead) All patients	Letter inviting patients for a lung health check including: - statement that smokers and ex-smokers are being invited - pre-scheduled appointment - contact details to cancel/rearrange/further information - information to help journey planning (map/address/stations/buses) Second copy of information booklet.	Control letter with one exception: - statement changed to say that people who have ever smoked are being invited (rather than smokers and ex-smokers specifically) Second copy of targeted information leaflet Brief essential information on the reverse side of the letter including details for requesting free copy of information booklet (phone or online)
Appointment	Run by Research Nurse All patients attending the appt	Information booklet (same as mailed previously). Nurse-led facilitation of informed decision-making	Identical to control
Reminder	Mailed ≥4 weeks after missed appt From GP (signature and letterhead) Patients who miss their appointment without cancelling	Letter re-inviting the patient for a lung health check appointment with similar content to the invitation letter	Control letter with one exception: - statement changed to say that people who have ever smoked are being invited (rather than smokers and ex-smokers specifically)

1. a pre-invitation letter notifying patients of the lung health check service and an information booklet mimicking so far as possible, those of existing screening programmes,
2. an invitation letter with a pre-scheduled appointment plus the same information booklet that accompanied the pre-invitation letter,
3. a reminder re-invitation letter for those who miss their appointment without cancelling (sent ≥4 weeks following the missed appointment).

### Intervention invitation approach: a targeted, stepped and low burden invitation strategy

The intervention group will receive the same stages of invitation materials as the control group. The two differences are: i) instead of the information booklet they will receive a targeted leaflet (see Additional file 1), and ii) the invitation and reminder letters will use indirect phrasing to say that smokers and ex-smokers are being invited. Together, these manipulations aim to deliver a targeted, stepped and low-burden approach to information provision prior to the appointment which, in principle, would be practically feasible to implement on a national scale. The group we are inviting will be far from homogeneous but as it is not feasible to ascertain each individual recipient’s characteristics prior to invitation, we are attempting to provide the best ‘one size fits all’ approach; inclusive enough to target a variety of different characteristics but also conservative, so as not to unnecessarily deter one group at the expense of another’s uptake. Materials have been tested during four patient and public engagement sessions to ensure acceptability and comprehensibility and reviewed by our multidisciplinary team (psychology, respiratory medicine, radiology, smoking cessation, and primary care) and community-academic partners from our qualitative phase of work informing the invitation design ([25]; full paper in prep).

#### Targeted component

This has been developed in response to what is known about the characteristics and beliefs of the target group from our own and existing research ([25–29]; full paper in prep). It aims to minimise fear (particularly of an expected diagnosis at screening which actually has a low probability), fatalism, stigma and blame around lung cancer by: i) emphasising a supportive and non-judgemental service, ii) providing a lay explanation for how early detection of lung cancer can work (using a diagram to illustrate that the lung is a treatable organ which need not be completely removed because early treatment can be focussed within a lobe), iii) acknowledging that the invited generation were previously not as informed of the risks of smoking, iv) avoiding mention of smoking, smoking cessation, and risk where possible at the invitation stage, v) emphasising the salience for older adults, and vi) normalising the offer so as to not implicate the reason for invitation as being that lung cancer is suspected or that the recipient is being singled out.

#### Stepped approach

This is guided by the Precaution Adoption Process Model (PAPM) which depicts different stages of awareness, engagement, decision-making and action for preventive health behaviours [30]. It is a useful framework from which to hypothesise at what stage different types of information could most effectively be communicated. Given that the target group are likely to have no prior awareness of lung cancer screening, the first contact is designed to provide a positive introduction to the service to engage them with the idea, without the pressure of yet needing to decide whether to attend. Previous research has shown that advance notification letters for bowel cancer screening which include a low level of information successfully increase participation [31], particularly among men from socioeconomically deprived backgrounds [32]. Written communication thereafter contains cues to action intended to minimise non-intentional factors that reduce participation (i.e. forgetting and procrastination). These include prescheduled appointments, maps with travel information, and for those who do not respond, reminder re-invitations, which have previously been shown to be effective [31–34].

#### Low burden level of information prior to the appointment

The materials have a relatively low level of information to promote consideration of the offer in a way that does not overwhelm or overburden. This takes account of the inherent challenges of communicating risk, uncertainty and overdiagnosis [35, 36]; the scientific uncertainty of estimates for lung cancer screening, its fast-moving evidence base, the application of population risk modelling to individual risk profiles, and new medical terminology [37], difficulties comprehending this information which are likely to be further exacerbated by the low levels of health literacy and numeracy anticipated for the low SES target group [38], and fear of lung cancer, which may influence receptivity to information and the ability to weigh up information rationally [39]. Increased ambiguity of information has been shown to confuse, raise suspicion and promote risk aversion among individuals with low numeracy and low optimism [40, 41]. Furthermore, recipients’ first impressions of the amount of information could be important for information engagement as perceived cognitive ease has been associated with more positive appraisal of the information content [42].

All these factors considered, it seemed appropriate to reduce the complexity of the information provided and the decision required by the individual to that of deciding whether to attend to discuss the tests. Free and easy access to further information before the appointment will be clearly signposted on invitation materials (for both groups). Once at the appointment, the nurse can provide a supported environment for the communication of complex information and can facilitate informed decision-making. The patient can then choose whether to have the tests the same day or a different day and the nurse will ensure they do not feel under pressure to decide either way.

#### Social marketing

The proposed approach, supporting evidence, and detailed draft content, were communicated to a social marketing team, who have used their expertise to creatively design engaging materials tailored for the target audience. The colour scheme and typography of the targeted leaflet is based on the brand identities of businesses that target low income customers. The images used are representative of a diverse population and range of ages, so as to reflect and engage the target audience. The leaflet uses a non-authoritarian conversational tone and includes quotes from our qualitative work to introduce a social presence to the information ([25]; full paper in prep).

### Methods of data collection and outcome measures

#### Demographics and smoking

Data on age, sex, ethnicity, smoking status and postcode will be extracted from primary care records by practice administrators for all patients identified as eligible and invited. Postcodes will be converted to Index of Multiple Deprivation (IMD) scores and ranks on site by a researcher from a spreadsheet within which identifiable data fields have been hidden. A pseudo-anonymised spreadsheet containing all these data will then be compiled and exported to a researcher independent of the identification process for randomisation and entry into the study database. While developing this protocol, we surveyed members of the public and patients about accessing this data prior to consent and none interviewed had any objections.

At the appointment, these data will be verified by a nurse who will take informed consent for the collection of any further data post-attendance. Further data collection will include information on attendees’ highest level of education (as an additional measure of SES) and measures of smoking behaviour and history. These will include current smoking status (self-reported and CO verified), usual number of cigarettes smoked daily, age started smoking, pack-year history, use of other nicotine and tobacco products (pipes, cigars, electronic cigarettes, waterpipes, smokeless tobacco) nicotine dependence (two item Heaviness of Smoking Index) [43] and quit confidence within the next six months.

#### Primary outcome

Uptake of the lung health check appointments will be recorded by the nurses running the lung health check appointments prior to consent. It will be measured by attendance because the outcome of interest is whether participants can be adequately engaged to consider lung cancer screening. The aim is to provide a realistic indication of uptake in a real-world clinical context. Recording attendance is already standard practice in a clinical context and knowing that participation is being recorded for research purposes would introduce observer bias and undermine the research question.

#### Secondary outcomes

To further explore interest and uptake of screening, willingness to be screened will be used as a proxy measure to gauge interest among those attending who are ineligible for a LDCT scan, and uptake of LDCT scans will be recorded among those eligible. Data on informed decision-making (i.e. objective and subjective knowledge, decisional conflict, decisional satisfaction) will also be collected at the appointment using a paper questionnaire. Items have been adapted from existing studies and measures, and low literacy scales have been chosen where available [44–46]. These measures will allow us to ensure the targeted invitation strategy does not compromise the ability of patients to make an informed decision about screening at their appointment. Scores on these measures will be compared by invitation group to ensure intervention participants achieve either similar or improved scores.

### Sample size

The target sample size is 2000 patients. This is based on an estimate that 35 % of patients in the control group will attend, similar to initial uptake of colorectal cancer screening (by FOBT) in London within the two most deprived IMD quintiles [47]. The aim is to achieve a 7 % improvement in uptake on the basis of similar previous research. Studies testing targeted ‘psycho-educational’ invitations have achieved a 5.9 % higher uptake of colorectal cancer screening (flexible sigmoidoscopy) in deprived areas [48] and an 11.8 % increase in FOBT participation [49]. Also, a 7 % increase would deliver clinically meaningful benefit if scaled to a national programme. With 2000 patients split equally into two groups, statistical power to carry out two-sided tests at the 5 % significance threshold is 90 %.

### Statistical analysis methods

#### Primary analysis

The researcher carrying out the analyses will be blinded to group allocation. Un-blinding will occur after the primary data analysis is complete and has been checked and verified by a second researcher. Chi square associations and multivariate logistic regression analyses will be carried out to compare uptake between the intervention and control groups. These analyses will take an intention-to-treat approach, including all patients identified and randomised. Due to the nature of this study, there should be no missing data for uptake.

#### Secondary analysis

Interaction terms will be used in regression models to investigate if there are differences in demographic and smoking-related predictors of uptake and if these are associated with invitation group. The demographic and smoking-related characteristics of attenders from each invitation group will also be compared with those of the overall invited group to further test for any biases in uptake and to elucidate figures which could be used to help gauge uptake by the high risk in the event of a national lung cancer screening programme.

Further analyses will be carried out to explore willingness to be screened, uptake of LDCT scans and informed decision-making outcomes (i.e. knowledge, decisional conflict, decisional satisfaction) by invitation group. This will function as a check that the intervention invitation materials do not adversely affect the patients’ ability to make an informed decision, given their low burden approach to information provision.

### Ethical approval, research governance and trial sponsorship

This study was approved by the City Road and Hampstead NHS Research Ethics Committee (REC; reference: 15/LO/1186) on the 29^th^ July 2015. Site-specific approval for the two hospital sites has been obtained via the Integrated Research Application System (IRAS), along with the necessary approvals from their Research and Development Departments. Any planned modifications to the protocol will be approved by the REC before they are adopted by the study.

This study has been adopted onto the NHS trial portfolio and is sponsored by University College London (UCL). The Joint Research Office (for UCL, UCH and the Royal Free) may carry out independent audits and on-site monitoring of the trial at any time and without notice; in adherence to UCL’s respective policies and the Department of Health’s Research Governance Framework for Health and Social Care.

### Study management

This study is a collaborative effort, run by the Health Behaviour Research Centre (HBRC) and the Lungs for Living (L4L) Research Centre. The trial management group (TMG) is comprised of the Principal Investigator, academic and clinical collaborators, and key researchers, who will together monitor trial conduct and progress. Data management, patient confidentiality and the conduct of all clinical and trial personnel will adhere to the full clinical trial protocol (version 2.0 or subsequent approved versions), Good Clinical Practice guidelines, essential standard operating procedures, the NHS Code of Confidentiality and the Data Protection Act (UCL Records Office registration number: Z6364106/2015/10/34). Inputting of data will comply with information governance legislation. An audit trail of documentation and data collection will be kept to enable monitoring by the research team and external regulatory bodies, and to protect against unintentional or unauthorised modification. Formal involvement of a Clinical Trials Unit (CTU) was deemed unnecessary by the UCL Institute of Clinical Trials and Methodology (ICTM) portal review group.

A Trial Steering Group (TSG) comprised of independent expert and lay members will meet with key members of the TMG to oversee this study and agree any amendments to the protocol. There will be meetings at six month intervals (approximately) throughout the trial recruitment phase. An Independent Data Monitoring Committee (IDMC) will review data on secondary clinical outcomes and sub-studies (to be reported elsewhere). There will be no interim review of the behavioural data as the behavioural intervention tested here poses minimal risk to patient safety.

### Trial status

This study began recruiting in October and is expected to recruit for 12 months.

## Discussion

This study will test a novel, low-cost and targeted invitation strategy for lung cancer screening, which aims to improve engagement with a screening offer by the high risk, especially low SES smokers. If shown to be effective, the materials and strategy could be translated for use by local screening pilots and a national screening programme were one to be implemented. The results would act as proof of principle that grass-roots research investigating psychosocial barriers to uptake within the local high risk community can effectively inform the development of engaging materials. Results will also inform the feasibility of inviting high risk patients to screening via primary care and provide figures to help estimate likely uptake of a screening programme. Findings from this study will be written in accordance with the CONSORT Statement [50], submitted for publication to relevant peer-reviewed journals and presented at conferences. A summary of results will provided to any participants who request this.

## Additional files

Additional file 1: Targeted information leaflet. (PDF 2.06 MB)

## Abbreviations

CCG: Clinical Commissioning Group
CO: carbon monoxide
CT: computed tomography
CTU: Clinical Trials Unit
FOBT: faecal occult blood test
GP: general practitioner
HBRC: Health Behaviour Research Centre
ICTM: Institute of Clinical Trials and Methodology
IDMC: Independent Data Monitoring Committee
IMD: Index of Multiple Deprivation
IRAS: Integrated Research Application System
L4L: Lungs for Living Research Centre
LDCT: low dose computed tomography
LLP: Liverpool Lung Project
NLST: National Lung Screening Trial
NSCLC: non-small cell lung cancer
PAPM: Precaution Adoption Process Model
REC: Research Ethics Committee
SES: socioeconomic status
TMG: Trial Management Group
TSG: Trial Steering Group
UCH: University College Hospital
UCL: University College London
UKLS: United Kingdom Lung Screening (trial)
USPSTF: United States Preventive Services Task Force
