# Supplementary material for: The Lung Screen Uptake Trial (LSUT): protocol for a randomised controlled demonstration lung cancer screening pilot testing a targeted invitation strategy for high risk and ‘hard-to-reach’ patients
Source: BMC Cancer. 2016 Apr 20;16:281. doi: 10.1186/s12885-016-2316-z (PMC4839109; doi:10.1186/s12885-016-2316-z)

APPENDIX 1: Targeted information leaflet: outer pages (tri-fold)

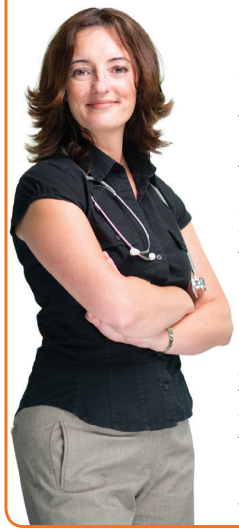

WHAT YOU'LL GET

First you'll be asked some questions about your breathing and how you feel to find out about your overall lung health.

Then by blowing into two hand-held machines, you'll be told whether there are any problems that need taking care of.

The nurse may also talk to you about having a lung scan to check for any early signs of lung cancer, and will ask if they can take samples of blood, breath, sputum and cheek cells (by rubbing a swab along the inside of the cheek). You can decide about this on the day or later.

You'll have plenty of time to chat to the nurse and ask any questions.

Bring a friend, family member or partner with you on the day if you want to.

"These lung checks are a brilliant idea - a great way to give hard-working lungs a service"

Bernie, Nurse
University College Hospital

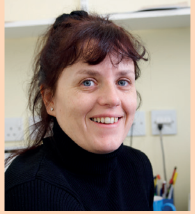

LUNG HEALTH CHECKS

GPs in the local area are inviting people aged 60 to 75 for the Lung Health Check.

Look out for an invitation in the post.

For more information call our freephone advice service on 0808 [redacted] or call/text 07469 [redacted] or email us at [redacted]

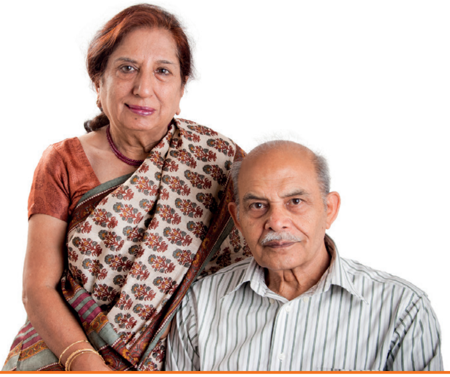

If you are unable to read this leaflet because English is not your first language, please ask someone who speaks English to telephone the Freephone helpline on **0800** [redacted] for further information and help.

Bengali ইংরেজী আপনার প্রথম বা মাতৃভাষা না হওয়ার কারণে আপনি যদি এই চিঠি বা সঙ্গে দেওয়া প্রচারপত্র পড়তে না পারেন, তাহলে ইংরেজী বলতে পারে এমন কাউকে বলুন আরো বিস্তারিত তথ্য ও সাহায্যের জন্য 0800 [redacted] নম্বরে ফ্রীফোন বা বিনা খরচের হেলপ্লাইন-এ টেলিফোন করতে।

Turkish İngilizce'nin anadiliniz olmaması nedeniyle bu mektubu veya ilişikteki broşürü okuyamayacak olursanız, daha fazla bilgi ve yardım için, lütfen, İngilizce bilen birisinden, ücretsiz olarak telefon edilebilen 0800 [redacted] numaralı yardım hattını aramasını rica edin.

NHS

M.O.T. FOR YOUR LUNGS

A new NHS Lung Health Check for people aged 60 to 75

Please read if you are aged 60-75

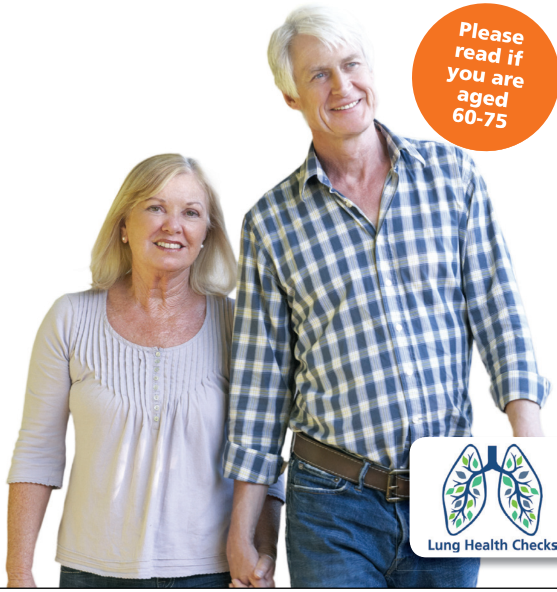

Lung Health Checks

APPENDIX 1: Targeted information leaflet: inner pages (tri-fold)

M.O.T. FOR YOUR LUNGS

People aged 60 to 75 are being offered a new LUNG HEALTH CHECK.

Run by specially trained nurses, they are an easy way to find out how well your lungs are working.

And, if needed, you'll get care and treatment to help breathe new life into your lungs.

The checks are for people aged 60 to 75 who have ever smoked. You are invited whether you feel fine or not, and whether or not you have any lung problems.

You can bring a friend, family member or partner with you, if you'd like.

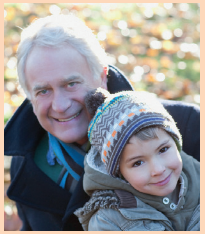

"If they can give me some extra years with my grandkids, I might even be lucky enough to be able to walk them down the aisle."

Bernard, 69, London

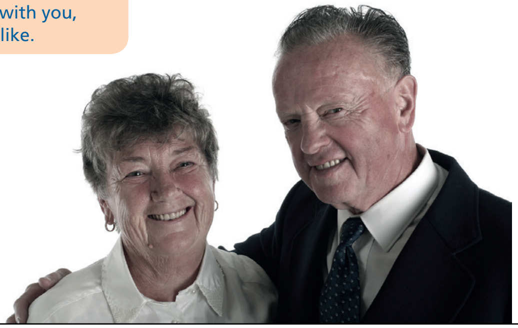

BENEFITS OF THE LUNG HEALTH CHECK

- ✓ **Free**
- ✓ **Local and easy to get to** (at either the Homerton or University College Hospital)
- ✓ **Talk through your questions** over a cup of tea
- ✓ **Find out** about having a lung scan
- ✓ **No judgements** on smoking

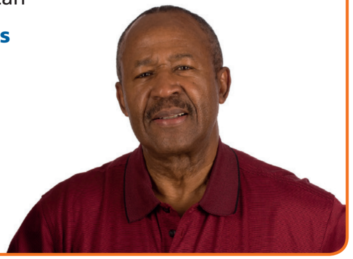

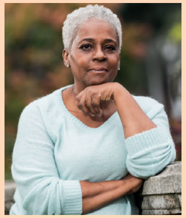

"I started smoking when I was 14. When you go back 40-odd years, we didn't know that cigarettes caused all these problems. It's good to know no one is going to give me a hard time at the Lung Health Check."

Maggie, 60, London

LOOKING AFTER YOUR LUNGS

HOW THE NHS CAN HELP

Your lungs work hard every minute of your life.

As you get older, it's worth checking things out.

GOOD IDEA

The Lung Health Check can spot problems early - often before you notice anything, when treatment could be simpler and more successful.

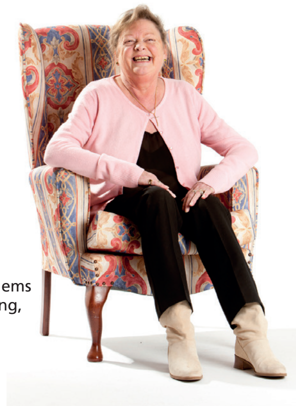

YOUR LUNGS COULD BE EASIER TO FIX THAN YOU THINK

You have two lungs, made up of 5 sections called lobes.

Each lobe is made up of thousands of tiny grape-like sacs, called alveoli.

If there is a problem on one bit of the lung, early treatment can focus just on the bit that is affected.

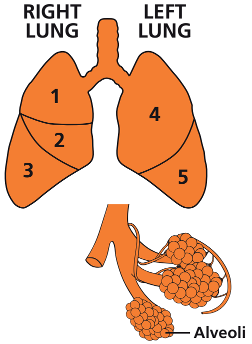

Supplement: Additional file 1: — Targeted information leaflet. (PDF 2.06 MB) [file 12885_2016_2316_MOESM1_ESM.pdf]
